# BMC Dermatology reviewer acknowledgement, 2012

**DOI:** 10.1186/1471-5945-13-4

**Published:** 2013-04-15

**Authors:** Hayley Henderson

**Affiliations:** 1 BioMed Central, Floor 6, 236 Gray's Inn Road, London WC1X 8HB, United Kingdom

## Abstract

**Contributing reviewers:**

The editors of *BMC Dermatology* would like to thank all our reviewers who have contributed to the journal in Volume 12 (2012).

## 

Ole Ahlehoff

Denmark

Ayadi Ali

Tunisia

Marco Calogero Amato

Italy

Giuseppe Argenziano

Italy

Benoît Arveiler

France

Andrzej Bieniek

Poland

Ivan Birch

United Kingdom

Anthony Bullock

United Kingdom

Ian Burgess

United Kingdom

John Burgess

Australia

Ali Cadili

Canada

Sergio Chimenti

Italy

Tadd Clayton

New Zealand

Arnon Cohen

Israel

Darren Day

New Zealand

Marília-Gerhardt De Oliveira

Brazil

Esther De Vries

Netherlands

Chukwuma Ekpebegh

South Africa

Nadia El Fekih

Tunisia

Brigitte Essers

Netherlands

John Foerster

United Kingdom

Asli Gelincik

Turkey

Anthony Goon

Singapore

Shoshana Greenberger

Israel

Metin Gungormus

Turkey

Melvin Hayden

USA

Masaru Honma

Japan

Nicholas Inston

United Kingdom

Slavenka Jankovic

Serbia

Jams Jayatilake

Sri Lanka

Myungshin Kim

Korea South

Jacob Levitt

USA

Richard Liebano

Brazil

Marie Loden

Sweden

Blazej Meczekalski

Poland

Kim Meeuwis

Netherlands

Elvira Moscarella

Italy

Luigi Naldi

Italy

Lill Tove Nilsen

Norway

Emeka Nweze

Nigeria

Monia Orciani

Italy

Sean Palecek

USA

Aldona Pietrzak

Poland

Olivier Piot

France

Rosa Maria Ponce

Mexico

Toshiaki Saida

Japan

Omar Sedrati

Morocco

Tor Shwayder

USA

Georgios Stamatas

France

Denise Steiner

Brazil

Joanna Stewart

New Zealand

Kursad Unluhizarci

Turkey

V Vijayanath

India

Sümer Yamaner

Turkey

## Pre-publication history

The pre-publication history for this paper can be accessed here:

http://www.biomedcentral.com/1471-5945/13/4/prepub

